# Reducing stiffness of shock-absorbing pylon amplifies prosthesis energy loss and redistributes joint mechanical work during walking

**DOI:** 10.1186/s12984-021-00939-8

**Published:** 2021-09-21

**Authors:** Jenny Anne Maun, Steven A. Gard, Matthew J. Major, Kota Z. Takahashi

**Affiliations:** 1 Department of Biomechanics, University of Nebraska at Omaha, Omaha, NE USA; 2 Department of Physical Medicine and Rehabilitation, Feinberg School of Medicine, Northwestern University, Chicago, IL USA; 3 Department of Biomedical Engineering, Northwestern University, Evanston, IL USA; 4 Jesse Brown VA Medical Center, Chicago, IL USA

**Keywords:** Amputation, Gait, Biomechanics, Power, Damping, Dissipation, Absorption

## Abstract

**Background:**

A shock-absorbing pylon (SAP) is a modular prosthetic component designed to attenuate impact forces, which unlike traditional pylons that are rigid, can compress to absorb, return, or dissipate energy. Previous studies found that walking with a SAP improved lower-limb prosthesis users’ comfort and residual limb pain. While longitudinal stiffness of a SAP has been shown to affect gait kinematics, kinetics, and work done by the entire lower limb, the energetic contributions from the prosthesis and the intact joints have not been examined. The purpose of this study was to determine the effects of SAP stiffness and walking speed on the mechanical work contributions of the prosthesis (i.e., all components distal to socket), knee, and hip in individuals with a transtibial amputation.

**Methods:**

Twelve participants with unilateral transtibial amputation walked overground at their customary (1.22 ± 0.18 ms^−1^) and fast speeds (1.53 ± 0.29 ms^−1^) under four different levels of SAP stiffness. Power and mechanical work profiles of the leg joints and components distal to the socket were quantified. The effects of SAP stiffness and walking speed on positive and negative work were analyzed using two-factor (stiffness and speed) repeated-measure ANOVAs (α = 0.05).

**Results:**

Faster walking significantly increased mechanical work from the SAP-integrated prosthesis (p < 0.001). Reducing SAP stiffness increased the magnitude of prosthesis negative work (energy absorption) during early stance (p = 0.045) by as much as 0.027 Jkg^−1^, without affecting the positive work (energy return) during late stance (p = 0.159), suggesting a damping effect. This energy loss was partially offset by an increase in residual hip positive work (as much as 0.012 Jkg^−1^) during late stance (p = 0.045). Reducing SAP stiffness also reduced the magnitude of negative work on the contralateral sound limb during early stance by 11–17% (p = 0.001).

**Conclusions:**

Reducing SAP stiffness and faster walking amplified the prostheses damping effect, which redistributed the mechanical work, both in magnitude and timing, within the residual joints and sound limb. With its capacity to absorb and dissipate energy, future studies are warranted to determine whether SAPs can provide additional user benefit for locomotor tasks that require greater attenuation of impact forces (e.g., load carriage) or energy dissipation (e.g., downhill walking).

**Supplementary Information:**

The online version contains supplementary material available at 10.1186/s12984-021-00939-8.

## Background

Daily activities, like walking, are common challenges experienced by individuals with a lower-limb amputation [1–5]. Individuals with an amputation seek a prosthesis that allows both mobility and comfort [5–7]. While considerable attention has been paid to studying the effects of prosthetic feet [8–10] and ankle components [11–18], relatively less is known regarding the effects of prosthetic pylons on mobility outcomes. A shock-absorbing pylon (SAP), in particular, is a modular component designed to attenuate impact forces to assist with weight acceptance [7, 19–21]. Unlike traditional pylons that rigidly connect from the socket to the distal foot and ankle components, SAPs comprise of a telescoping shank with viscoelastic properties that can compress [19, 22, 23] and/or twist about the longitudinal axis [24, 25]. Prior studies have suggested that wearing a prosthesis with a SAP can improve user comfort and reduce residual limb pain compared to walking with a rigid pylon [19, 22]. However, there is currently limited knowledge regarding how SAPs directly affect the prostheses’ ‘shock-absorbing’ capabilities—in particular, the mechanical work done on the prosthesis which relates to the energy absorption, storage, and/or return during walking.

Mechanical work done on the SAP is influenced by the forces acting on the pylon and its stiffness, which affects the SAP’s longitudinal displacement. Prior studies involving SAPs have revealed their capacity for influencing the forces transmitted to the legs during walking [19, 22]. For example, walking with a SAP reduced ground reaction force magnitudes during early stance, especially at speeds greater than 1.3 ms^−1^ [19, 22]. Such force attenuation may theoretically influence energy absorption during early stance (also termed ‘collision work’), which relates to the mechanics of the leading leg as weight is being transferred from the trailing leg [26]. However, the work done by SAP-integrated prostheses have not been directly quantified. While the anatomical ankle–foot structures have natural shock-absorbing mechanisms, such as the heel pad [27–29] and ankle muscles [30, 31], it is currently unclear whether the SAPs contribute directly to energy absorption during collision.

A recent study analyzed the work of the center-of-mass (or individual limbs) when participants walked with SAPs of various stiffness [32]. Results from that study suggested reducing pylon stiffness increased the magnitude of prosthetic limb negative work during early stance (i.e., collision work) and enhanced prosthetic limb positive work during late stance (i.e., push-off work) [32]. While this study established the SAP’s potential to affect the work done on the center-of-mass, the relative work contributions of the prosthesis and the intact joints remain unknown. Partitioning the work contributions may reveal additional insights regarding the interaction between the user and the prosthesis—specifically, how changes in SAP stiffness directly affect prosthesis energetics and indirectly affect compensations at the intact joints. For example, prior studies in prosthetic foot and ankle components have revealed that the energetics of the prostheses can directly affect ipsilateral or residual hip joint kinematics and kinetics [33–35] and collision work on the contralateral sound limb [9, 11]. Thus, quantifying work contributions during various phases of stance within and across the lower limbs, including the prosthesis components and the intact joints, is expected to reveal greater insights about the effects of SAP-integrated prostheses on walking outcomes.

The purpose of our study was to determine the effect of SAP stiffness and walking speed on the mechanical work contributions from the prosthesis (i.e., components distal to the socket), proximal intact joints (residual knee and hip), and the contralateral sound limb of individuals with a unilateral transtibial amputation. Based on similar studies involving prosthetic ankle stiffness [9, 16, 33], we hypothesized that reducing SAP stiffness would increase the magnitude of negative work during early stance and positive work in late stance within the prosthesis—which would indicate greater energy stored and returned, respectively. Assuming that the mechanical work within a leg is conserved (i.e., the net work is near zero during steady-state walking), we expected the proximal leg joints to act in opposition to the prosthesis behavior. We hypothesized that reducing SAP stiffness would decrease the residual knee and hip joints’ magnitudes of negative work during early stance and positive work during late stance. Lastly, we hypothesized that faster walking speed will reveal greater effects of stiffness on the prosthesis, residual knee, and residual hip work to correspond with increased ground reaction forces.

## Methods

### Participants

A secondary analysis was performed on a dataset from a previous study [23] that included 12 participants with unilateral transtibial amputation (4 females/8 males; age: 48.9 ± 17.9 years; height: 1.80 ± 0.10 m; mass: 84.8 ± 21.0 kg; time since amputation: 12.8 ± 8.9 yrs). All participants had at least six months of experience walking with their current prosthesis and were able to walk independently without assistance for at least 10 m. All participants provided written, informed consent to participate. Northwestern University Institutional Review Board approved this study.

### Protocol

In the original study from Boutwell et al. [23], participants walked overground along a 10 m level walkway at two self-paced walking speeds (customary—1.22 ± 0.18 and fast—1.53 ± 0.29 ms^−1^; means ± SD) under four stiffness conditions: SOFT, MED, NORM, and RIGID. Participants walked at both speeds while first wearing the NORM stiffness as the control condition to obtain a baseline speed. The rest of the stiffness conditions were randomized and walking speed was controlled through instantaneous measurement and verbal feedback to be within ± 10% of their baseline walking speed. Participants walked back and forth along a 10 m walkway until 5 clean force plate strikes were obtained from both limbs, defined as a single foot landing within the boundaries of a single force plate (AMTI, Watertown, MA). More detailed information on the experimental protocol can be found in the Boutwell study [23]. Participants walked in their prescribed socket and suspension system, while all components distal to the socket (i.e., the experimental prosthesis) were the same for all participants during the experiment. The components of the experimental prosthesis were the SAP (Endolite Telescoping Torsion Pro; Endolite, Miamisburg, OH) and a standard prosthetic foot (Seattle Lightfoot; Trulife, Dublin, Ireland) inserted into flat shoes with minimal sole thickness to control and minimize footwear effects [36]. The Endolite Telescoping Torsion Pro SAP was equipped with interchangeable springs to modify the longitudinal stiffness of the pylon. The torsion, or twisting, of the SAP (i.e., transverse motion) was disabled to focus on the pylon’s longitudinal stiffness. Prostheses were fitted and aligned by a certified prosthetist. Participants were given at least five minutes of walking to familiarize to each prosthesis configuration.

All stiffness conditions were a percentage of the manufacturer-recommended NORM stiffness: SOFT (50% of NORM), MED (75% of NORM stiffness), and RIGID (above NORM stiffness (Table 1) [23, 32]. The RIGID stiffness comprised of a steel rod inside the SAP instead of a spring to stop pylon displacement. One spring set was assigned to a participant by associating the NORM stiffness based on their body mass and activity level, with spring set 2 slightly stiffer than spring set 1. Five participants were assigned to spring set 1 (mass: 71.9 ± 5.04 kg; means ± SD) and seven participants were assigned to spring set 2 (mass: 99.1 ± 19.1 kg).

**Table 1:** The four stiffness conditions and their values for each spring set were measured by mechanical testing from Boutwell et al. [23]

Stiffness condition	SOFT (kNm^−1^)	MED (kNm^−1^)	NORM (kNm^−1^)	RIGID (kNm^−1^)	Average mass (kg)
Spring set 1	68.2	89.3	111.8	3556.9	71.9 ± 5.04
Spring set 2	85.6	111.8	153.8	3556.9	99.1 ± 19.1

### Data analysis

A modified Helen Hayes full-body marker set [37] with 24 markers were applied. Lower-limb kinematic and kinetic data were collected using a 12-camera motion capture system (Motion Analysis Corporation, Rohnert Park, CA) at 240 Hz and 6 floor-embedded force plates (Advanced Mechanical Technology, Inc. Watertown, MA) captured at 1920 Hz. Kinematic and kinetic data were processed using Visual3D (C-Motion, Inc., Germantown, MD) and were filtered using a low-pass Butterworth filter with a frequency cut-off of 6 Hz and 25 Hz, respectively. The analysis was performed on all available foot strikes for each stiffness and speed condition, verified visually in Visual3D through foot contact within the border of a single force plate and via analyses of center-of-pressure location relative to the foot’s geometry.

**Fig. 1:**
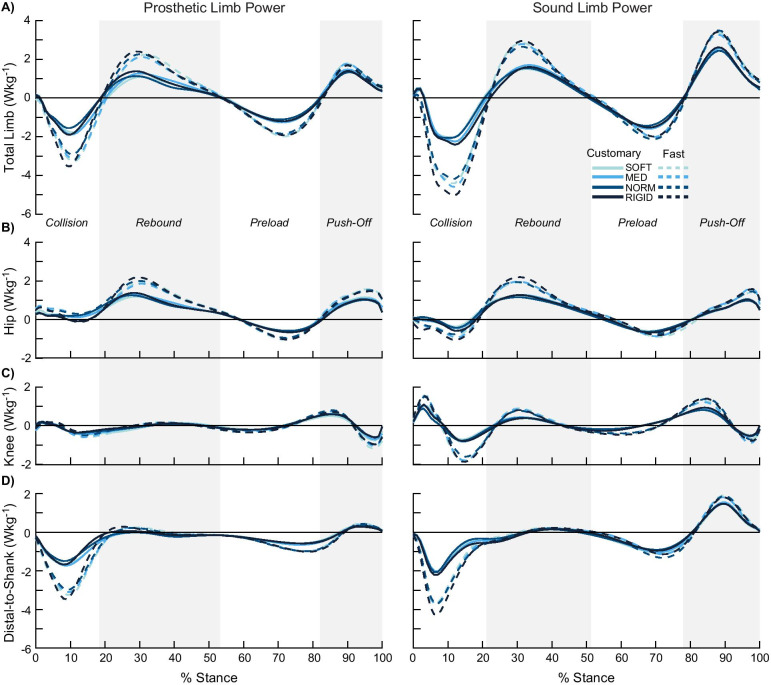
Lower limb power. Average time-series of power (Wkg^−1^) during stance phase (n = 12) were computed for each stiffness (light blue = SOFT to dark blue = RIGID) and speed (solid line = customary speed, dotted line = fast speed). Sub-phases (collision, rebound, preload, and push-off) are divided by gray panels and determined when total limb power crosses zero. Prosthesis limb power is on the left column and sound limb power is on the right column. Total limb power (**A**) is the sum of the powers at the hip (**B**), knee (**C**), and prosthesis or distal-to-shank (**D**)

Mechanical powers (Wkg^−1^) from all prosthetic components distal to the socket (i.e., the prosthesis) and ankle–foot complex of the sound limb were calculated using the unified deformable power analysis [38]. Knee and hip joint power from both limbs were calculated using a six degree-of-freedom joint power analysis, which takes into account the three degrees of rotation and the three degrees of translation about a joint [39, 40]. A prior study indicated that including all six degree-of-freedom improves the accuracy of the total work done on the whole-body, compared to a more traditional three degree-of-freedom analysis [40]. Total limb power was estimated as the sum of the prosthesis or sound limb ankle–foot complex, knee, and hip power, which captured the rate of energy change at the center-of-mass [40]. The time-series profile of the total limb power was used to identify four sub-phases of stance [40, 41] where the start and end of each sub-phase was defined as the instance when total limb power was zero, or crossed the x-axis (Fig. 1). The sub-phases were defined as: (1) collision—negative power and work during early stance, (2) rebound—positive power and work during early-to-mid stance, (3) preload—negative power and work during mid-to-late stance, and (4) push-off—positive power and work during late stance.

Positive, negative, and net mechanical work (Jkg^−1^) of the prosthesis, ankle–foot complex, knee, hip, and total limb across entire stance and each sub-phase (determined by the total limb power) were calculated by integrating power with respect to time using custom MATLAB code (Mathworks, Natick, MA). All kinetic variables (power and work) were normalized by body mass (kg).

### Prosthesis analysis

To further partition the contributions from the prosthesis components, we quantified the work done by the pylon compression. Power from pylon compression was calculated as the product between the force along the longitudinal axis (i.e., ground reaction force transformed from the laboratory to shank’s coordinate system [42]) and the compression velocity. The compression velocity of the pylon was computed as the derivative of the pylon’s displacement, or the length of the pylon during the entire stance phase. The length of the pylon was calculated using the distance formula between two points in a 3D coordinate system. The two markers were placed on the front-facing portion of the pylon: at the top of the pylon (located just below the prosthetic socket) and at the bottom of the pylon (located just above the prosthetic foot at the “ankle” location). About 25 cm of space was needed between the socket and the ground to fit the pylon and prosthetic foot [23]. Work from the pylon compression was quantified as the time-integral of the compression power during the entire stance phase. The power contribution due to prosthetic twisting was not included in the analysis as the transverse motion of the pylon was disabled. It is important to note that the prosthesis analyses did not account for the residuum-socket interface.

### Statistical analysis

Two-factor (stiffness and speed) repeated-measures analysis of variance (ANOVA) assessed the main effects of SAP stiffness and walking speed on the positive and negative mechanical work variables of both prosthetic and sound legs (prosthesis or ankle–foot complex, knee, hip, and total limb), with the critical *α* of 0.05. The two-factor ANOVA also assessed the interaction effects between stiffness and speed, to determine whether the influence of SAP stiffness on mechanical work varied between the two self-paced walking speeds. Data normality and sphericity was confirmed using the Shapiro–Wilk and Mauchly tests, respectively. A Greenhouse–Geisser correction was implemented when sphericity was violated. If the main effect of stiffness was significant, multiple post-hoc pairwise comparisons were conducted with a Bonferroni correction to account for Type-I error risk. If the interaction effects were significant, the simple main effect of stiffness was assessed by calculating the estimated marginal means of each stiffness while holding each speed constant using a one-factor (stiffness) ANOVA. Additionally, two-factor (stiffness and speed) repeated-measures ANOVA were used to assess the main and interaction effects of SAP stiffness and walking speed on the positive and negative mechanical work of the prosthesis due to pylon compression, with a critical *α* of 0.05. Statistical analyses were conducted using SPSS (IBM, Chicago, IL). Due to the exploratory nature of our analyses, we did not perform any *α* level adjustments as a result of multiple hypotheses testing. However, we reported all p-values and effect sizes (computed as partial eta squared, *η*^2^_*p*_) for full transparency of the results.

## Results

### Total prosthetic limb (prosthesis + knee + hip)

**Fig. 2:**
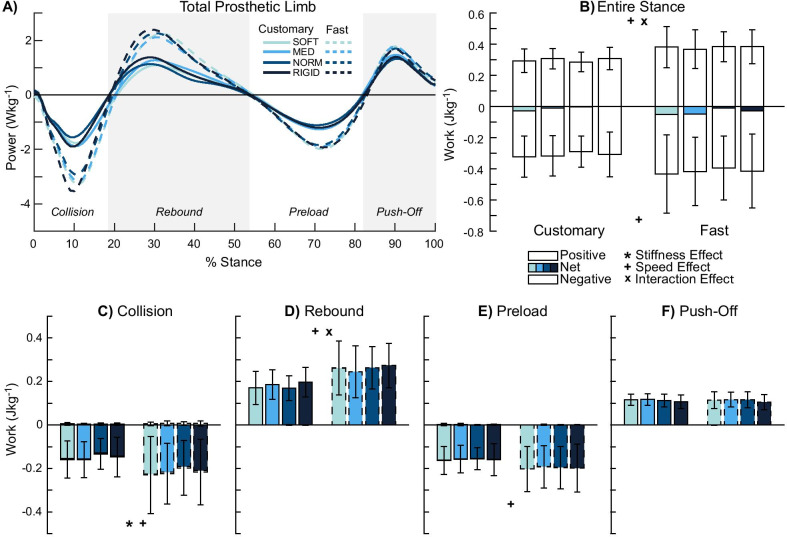
Total prosthetic limb power and work. Average power by the total prosthetic limb (n = 12) (**A)** and mechanical work were computed in the entire stance (**B**) and during each of the four sub-phases: collision (**C**), rebound (**D**), preload (**E**), and push-off (**F**). Collision (**C**) showed a stiffness effect. Faster walking speed produced greater work in the entire stance phase (**B**) and most sub-phases (**C**–**E**). Positive stance work (**B**) showed an interaction effect. Overall, the total prosthetic limb produced near zero net work in the entire stance phase (**B**). Significant stiffness, speed, and interaction effects are denoted by ‘*’, ‘ + ’, and ‘x’ symbols, respectively

There were no stiffness effects on positive and negative work in the entire stance phase (p ≥ 0.115, *η*^2^_*p*_ ≤ 0.162, Fig. 2A, B). While the rebound (p ≥ 0.056, *η*^2^_*p*_ ≤ 0.202, Fig. 2D), preload (p ≥ 0.635, *η*^2^_*p*_≤0.050, Fig. 2E), and push-off (p ≥ 0.058, *η*^2^_*p*_ ≤ 0.204, Fig. 2F) sub-phases were not significant, negative collision work showed a stiffness effect (p = 0.041, *η*^2^_*p*_ = 0.219, Fig. 2C). There were no significant post-hoc pairwise comparisons for negative collision work (p ≥ 0.152).

There were speed effects for both positive and negative work in the entire stance phase (p ≤ 0.005, *η*^2^_*p*_ ≤ 0.668, Fig. 2B). Fast walking was associated with increased stance work: 27% greater positive and 34% greater negative stance work compared to customary walking. Apart from the push-off sub-phase (p ≥ 0.473, *η*^2^_*p*_ ≤ 0.048), all other sub-phases showed speed effects (p ≤ 0.019). Fast walking was also associated with increased sub-phase work: 45% greater negative collision work (p = 0.006, *η*^2^_*p*_ = 0.511), 44% greater positive rebound work (p < 0.001, *η*^2^_*p*_ = 0.686), and 24% greater negative preload work (p = 0.019, *η*^2^_*p*_ = 0.405) compared to customary walking.

There was an interaction effect for only positive work in the entire stance phase (p = 0.009, *η*^2^_*p*_ = 0.293). Simple main effects analysis for positive stance work revealed no stiffness effect (p ≥ 0.92, *η*^2^_*p*_ ≤ 0.005); however, speed effects were observed in SOFT (p = 0.026, *η*^2^_*p*_ = 0.055) and NORM (p = 0.013, *η*^2^_*p*_ = 0.068) stiffness conditions, where fast walking led to 30–35% greater positive stance work. While the collision (p ≥ 0.167, *η*^2^_*p*_ ≤ 0.152), preload (p ≥ 0.347, *η*^2^_*p*_ ≤ 0.083), and push-off (p ≥ 0.325, *η*^2^_*p*_ ≤ 0.089) sub-phases had non-significant interaction effects, this effect was significant for positive rebound work (p = 0.019, *η*^2^_*p*_ = 0.311). Simple main effects analysis for positive rebound work revealed no stiffness effect (p ≥ 0.87, *η*^2^_*p*_ ≤ 0.008); however, speed effects were observed in SOFT (p = 0.017, *η*^2^_*p*_ = 0.063), NORM (p = 0.015, *η*^2^_*p*_ = 0.066), and RIGID (p = 0.043, *η*^2^_*p*_ = 0.046) stiffness conditions, where fast walking was associated with 39–56% greater positive rebound work.

### Prosthesis (distal to the socket)

**Fig. 3:**
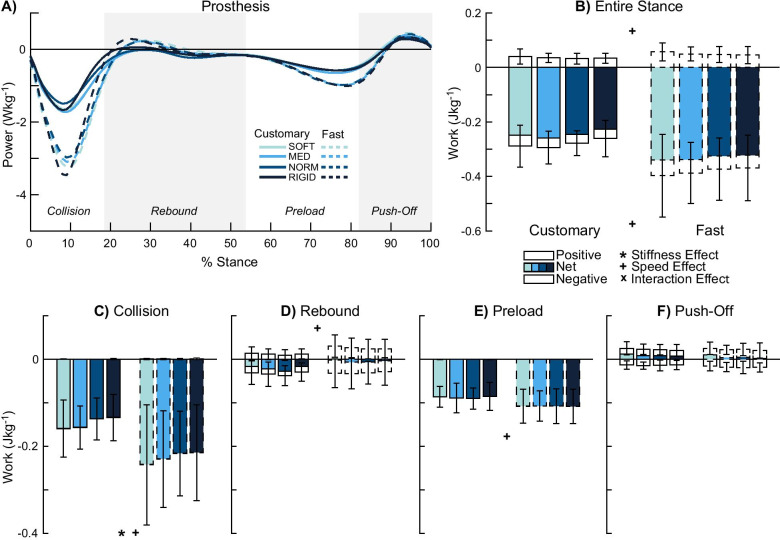
Prosthesis (distal to socket) power and work. Average power by the prosthesis (n = 12) (**A**) and mechanical work were computed in the entire stance (**B**) and during each of the four sub-phases: collision (**C**), rebound (**D**), preload (**E**), and push-off (**F**). Collision (**C**) showed a stiffness effect. Faster walking speed produced greater work in the entire stance phase (**B**) and most sub-phases (**C**–**E**). Overall, the prosthesis produced net negative work in the entire stance phase (**B**). Significant stiffness, speed, and interaction effects are denoted by ‘*’, ‘ + ’, and ‘x’ symbols, respectively

There were no stiffness effects on positive and negative work in the entire stance phase (p ≥ 0.192, *η*^2^_*p*_ ≤ 0.329, Fig. 3A, B). Apart from the rebound (p ≥ 0.236, *η*^2^_*p*_ ≤ 0.119, Fig. 3D), preload (p ≥ 0.426, *η*^2^_*p*_ ≤ 0.067, Fig. 3E), and push-off (p ≥ 0.159, *η*^2^_*p*_ ≤ 0.143, Fig. 3F) sub-phases, negative collision work showed a stiffness effect (p = 0.045, *η*^2^_*p*_ = 0.212, Fig. 3C). There were no significant post-hoc pairwise comparisons for negative collision work (p ≥ 0.163).

There were speed effects for both positive and negative work in the entire stance phase (p ≤ 0.017, *η*^2^_*p*_ ≤ 0.592). Fast walking was associated with increased stance work: 43% greater positive and 36% greater negative stance work compared to customary walking. All sub-phases apart from the push-off phase (p ≥ 0.075, *η*^2^_*p*_ ≤ 0.260), showed speed effects (p ≤ 0.017). Fast walking was also associated with increased sub-phase work: 55% greater negative collision work (p = 0.002, *η*^2^_*p*_ = 0.612), 117% greater positive rebound work (p = 0.004, *η*^2^_*p*_ = 0.537), and 24% greater negative preload work (p = 0.010, *η*^2^_*p*_ = 0.466) compared to customary walking.

There were no interaction effects in the entire stance phase (p ≥ 0.791, *η*^2^_*p*_ ≤ 0.031) and sub-phases (p ≥ 0.389, *η*^2^_*p*_ ≤ 0.080).

### Residual knee

**Fig. 4:**
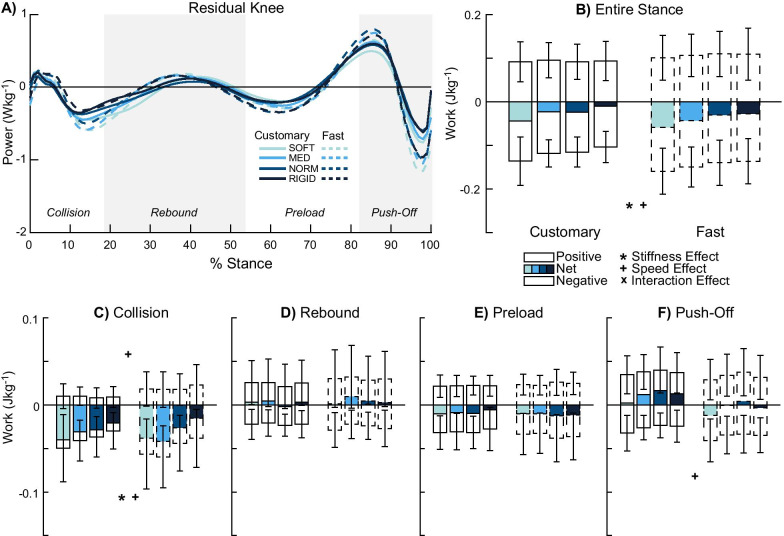
Residual knee power and work. Average power by the residual knee (n = 12) (**A**) and mechanical work were computed in the entire stance (**B**) and during each of the four sub-phases: collision (**C**), rebound (**D**), preload (**E**), and push-off (**F**). Entire stance (**B**) and collision (**C**) showed stiffness effects. Faster walking speed produced greater work in the entire stance phase (**B**) and during collision (**C**) and push-off (**F**). Overall, the prosthetic limb’s knee produced net negative work in the entire stance phase (**B**). Significant stiffness, speed, and interaction effects are denoted by ‘*’, ‘ + ’, and ‘x’ symbols, respectively

There was a stiffness effect for only negative work in the entire stance phase (p = 0.03, *η*^2^_*p*_ = 0.312, Fig. 4A, B). One post-hoc pairwise comparison was significant (SOFT-NORM p = 0.025), where reduced stiffness produced 16% greater negative work during the entire stance phase. The rebound (p ≥ 0.345, *η*^2^_*p*_ ≤ 0.094, Fig. 4D), preload (p ≥ 0.585, *η*^2^_*p*_ ≤ 0.043, Fig. 4E), and push-off (p ≥ 0.097, *η*^2^_*p*_ ≤ 0.172, Fig. 4F) sub-phases did not have a significant stiffness effect, but it was significant for negative collision work (p = 0.001, *η*^2^_*p*_ = 0.395, Fig. 4C). Two post-hoc pairwise comparisons were significant (MED-NORM p = 0.007, MED-RIGID p = 0.007) where reduced stiffness produced 25–47% more negative collision work.

There was a speed effect for only negative work in the entire stance phase (p = 0.006, *η*^2^_*p*_ = 0.505). Fast walking was associated with 24% greater negative stance work compared to customary walking. Apart from the rebound (p ≥ 0.167, *η*^2^_*p*_ ≤ 0.166) and preload (p ≥ 0.22, *η*^2^_*p*_ ≤ 0.133) sub-phases, positive and negative collision work and negative push-off work showed speed effects (p ≤ 0.041). Fast walking was also associated with increased sub-phase work: 111% greater positive collision (p = 0.016, *η*^2^_*p*_ = 0.423) and 28% greater negative collision (p = 0.041, *η*^2^_*p*_ = 0.327) work and 33% greater negative push-off work (p = 0.016, *η*^2^_*p*_ = 0.426) compared to customary walking.

There were no interaction effects in the entire stance phase (p ≥ 0.584, *η*^2^_*p*_ ≤ 0.056) and sub-phases (p ≥ 0.184, *η*^2^_*p*_ ≤ 0.135).

### Residual hip

**Fig. 5:**
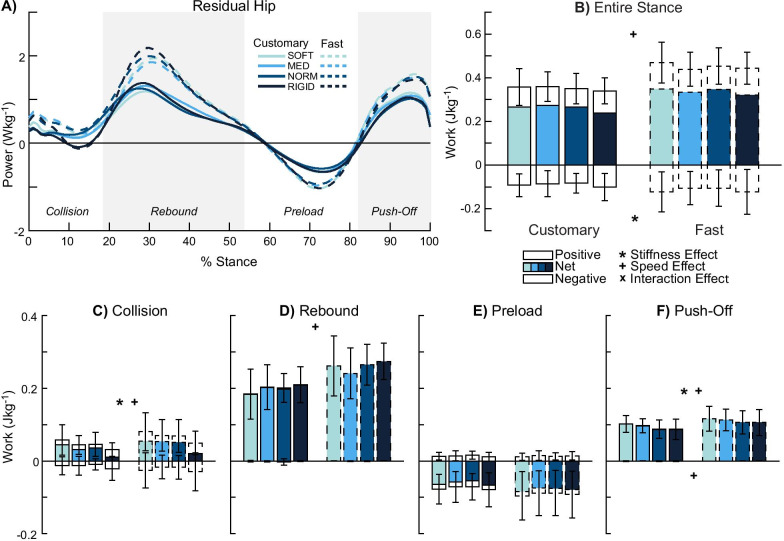
Residual hip power and work. Average power by the residual hip (n = 12) (**A**) and mechanical work were computed in the entire stance (**B**) and during each of the four sub-phases: collision (**C**), rebound (**D**), preload (**E**), and push-off (**F**). Entire stance (**B**), collision (**C**), and push-off (**F**) showed stiffness effects. Faster walking speed produced greater work in the entire stance phase (**B**) and most sub-phases (**C**–**D**, **F**). Overall, the prosthetic limb’s hip produced net positive work in the entire stance phase (**B**). Significant stiffness, speed, and interaction effects are denoted by ‘*’, ‘ + ’, and ‘x’ symbols, respectively

There was a stiffness effect for only negative work in the entire stance phase (p = 0.007, *η*^2^_*p*_ = 0.305, Fig. 5A, B). There were no significant post-hoc pairwise comparisons (p ≥ 0.065) for negative stance work. While the rebound (p ≥ 0.238, *η*^2^_*p*_ ≤ 0.118, Fig. 5D) and preload (p ≥ 0.172, *η*^2^_*p*_ ≤ 0.139, Fig. 5E) sub-phases were not significant, positive collision (p < 0.001, *η*^2^_*p*_ = 0.558, Fig. 5C) and positive push-off (p = 0.045, *η*^2^_*p*_ = 0.214, Fig. 5F) work showed stiffness effects (p ≤ 0.045). Four post-hoc pairwise comparisons were significant (SOFT-MED p = 0.044, SOFT-RIGID p = 0.005, MED-RIGID p = 0.006, NORM-RIGID p = 0.019), where reduced stiffness was associated with 21–73% greater positive collision work. However, there were no significant post-hoc pairwise comparisons for positive push-off work (p ≥ 0.158).

There were speed effects for only positive work in the entire stance phase (p < 0.001, *η*^2^_*p*_ = 0.703). Fast walking was associated with 28% greater positive stance work compared to customary walking. Apart from the preload sub-phase (p ≥ 0.163, *η*^2^_*p*_ ≤ 0.169), all other sub-phases showed speed effects (p ≤ 0.015). Fast walking was also associated with increased positive sub-phase work: 49% greater positive collision work (p = 0.007, *η*^2^_*p*_ = 0.503), 31% greater positive rebound work (p = 0.001, *η*^2^_*p*_ = 0.641), and 17% greater positive push-off work (p = 0.015, *η*^2^_*p*_ = 0.429) compared to customary walking. However, fast walking also associated with less negative work: 94% less negative push-off work (p = 0.045, *η*^2^_*p*_ = 0.317) compared to customary walking.

There were no interaction effects in the entire stance phase (p ≥ 0.348, *η*^2^_*p*_ ≤ 0.091) and sub-phases (p ≥ 0.13, *η*^2^_*p*_ ≤ 0.155).

### Total sound limb (ankle–foot + knee + hip)

**Fig. 6:**
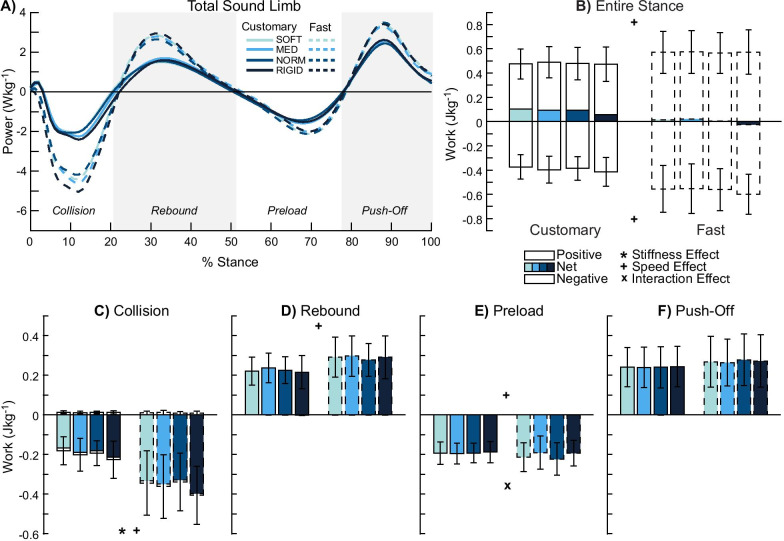
Total sound limb power and work. Average power by the total sound limb (n = 12) (**A**) and mechanical work were computed in the entire stance (**B**) and during each of the four sub-phases: collision (**C**), rebound (**D**), preload (**E**), and push-off (**F**). Entire stance (**B**) and collision (**C**) showed stiffness effects. Faster walking speed produced greater work in the entire stance phase (**B**) and most sub-phases (**C**–**E**). Preload (**E**) showed an interaction effect. Overall, the total sound limb produced net positive work in the entire stance phase (**B**). Significant stiffness, speed, and interaction effects are denoted by ‘*’, ‘ + ’, and ‘x’ symbols, respectively

There were no stiffness effects in the entire stance phase (p ≥ 0.057, *η*^2^_*p*_ ≤ 0.201, Fig. 6A, B). While the rebound (p ≥ 0.240, *η*^2^_*p*_ ≤ 0.118, Fig. 6D), preload (p ≥ 0.123, *η*^2^_*p*_ ≤ 0.159, Fig. 6E), and push-off (p ≥ 0.307, *η*^2^_*p*_ ≤ 0.095, Fig. 6F) sub-phases were not significant, negative collision work showed a stiffness effect (p = 0.001, *η*^2^_*p*_ = 0.396, Fig. 6C). Three post-hoc pairwise comparisons were significant (SOFT-RIGID p = 0.021, MED-RIGID p = 0.039, NORM-RIGID p = 0.01), where reduced stiffness was associated with 11–17% less negative collision work.

There were speed effects for both positive and negative work in the entire stance phase (p ≤ 0.001, *η*^2^_*p*_ ≤ 0.742). Fast walking was associated with 19% positive and 44% negative stance work compared to customary walking. Apart from the push-off sub-phase (p ≥ 0.079, *η*^2^_*p*_ ≤ 0.255), all other sub-phases showed speed effects (p < 0.001). Fast walking was also associated with increased sub-phase work: 81% greater negative collision work (p = 0.001, *η*^2^_*p*_ = 0.749), 28% greater positive rebound work (p = 0.001, *η*^2^_*p*_ = 0.642), and 69% greater positive preload work (p = 0.001, *η*^2^_*p*_ = 0.642) compared to customary walking.

There were no interaction effects (p ≥ 0.804, *η*^2^_*p*_ ≤ 0.029) in the entire stance phase. While the collision (p ≥ 0.238, *η*^2^_*p*_ ≤ 0.118), rebound (p ≥ 0.244, *η*^2^_*p*_ ≤ 0.117), and push-off (p ≥ 0.245, *η*^2^_*p*_ ≤ 0.121) sub-phases were not significant, negative preload work showed an interaction effect (p = 0.047, *η*^2^_*p*_ = 0.211). Simple main effects analysis revealed there were no stiffness (p ≥ 0.567, *η*^2^_*p*_ ≤ 0.023) and speed effects (p ≥ 0.256, *η*^2^_*p*_ ≤ 0.015) for negative preload work.

Work profiles of the sound ankle–foot (Additional file 1, Figure S1), knee (Additional file 2, Figure S2), hip (Additional file 3, Figure S3), and their written results (Additional file 4) are included as online additional material.

### Prosthesis analysis

**Fig. 7:**
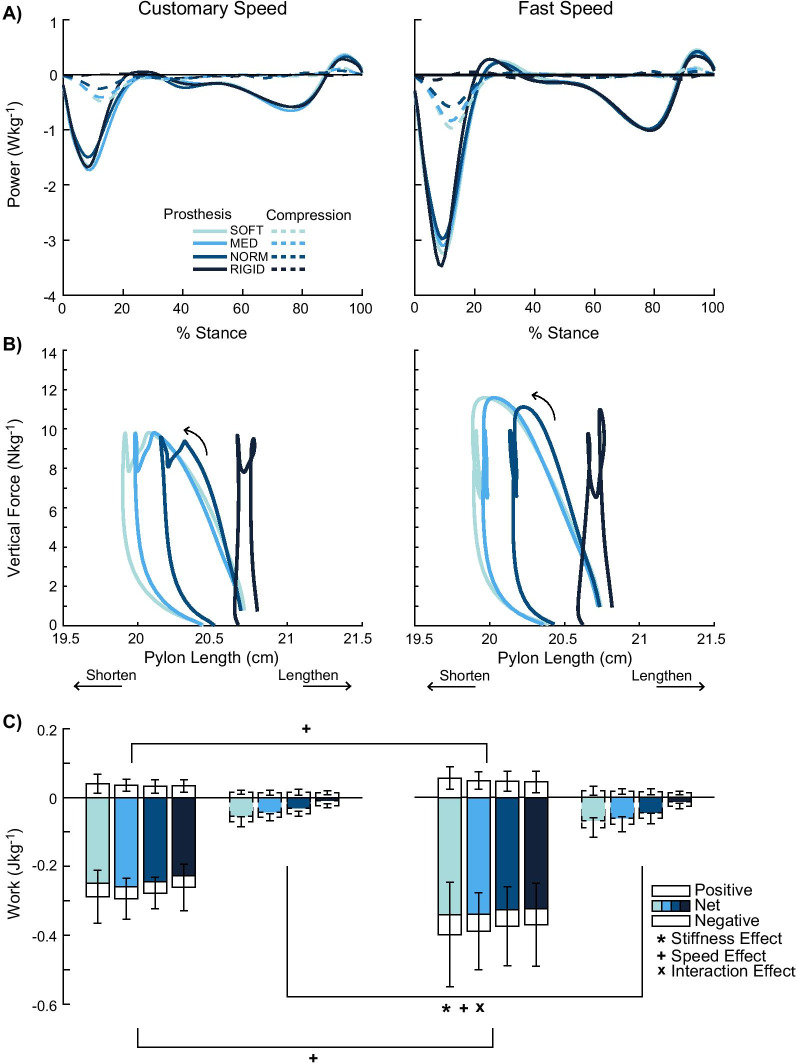
Prosthesis analysis. Additional analysis examined the average power (**A**), force–displacement profiles (**B**), and mechanical work in the entire stance phase (**C**) of the prosthesis and pylon compression in all levels of stiffness and speed. The force–displacement profiles (**B**) provide a visual representation of the net negative work produced from the pylon’s compression (arrows indicate the time-progression during stance). The magnitude of net negative work increased as the pylon became less stiff. Significant stiffness, speed, and interaction effects are denoted by ‘*’, ‘ + ’, and ‘x’ symbols, respectively

Additional analysis in the entire stance phase investigated the contribution of the pylon compression to the overall prosthetic work. There was a stiffness effect for pylon compression negative work during the entire stance phase (p < 0.001, *η*^2^_*p*_ = 0.907, Fig. 7C). All six post-hoc pairwise comparisons were significant (p ≤ 0.001), where reduced stiffness was associated with 18–229% more negative work.

There was also a speed effect for pylon compression negative work (p = 0.004, *η*^2^_*p*_ = 0.541), where fast walking increased negative work by 29% compared to customary walking. Pylon compression negative work also showed an interaction effect (p = 0.018, *η*^2^_*p*_ = 0.353). Simple main effects analysis revealed stiffness effects for both walking speeds (p < 0.001, *η*^2^_*p*_ ≤ 0.559). Four pairwise comparisons were significant at each speed (SOFT-NORM p = 0.003, SOFT-RIGID p < 0.001, MED-RIGID p < 0.001, and NORM-RIGID p = 0.003), where reduced stiffness was associated with 52–204% more negative work at customary walking and 44–252% more negative work at fast walking. Furthermore, simple main effects analysis for revealed speed effects in SOFT (p = 0.006, *η*^2^_*p*_ = 0.083), MED (p = 0.002, *η*^2^_*p*_ = 0.101), and NORM (p = 0.029, *η*^2^_*p*_ = 0.053) stiffness conditions, where fast walking was associated with 25–35% greater negative work.

A majority of the pylon compression’s negative net work in the entire stance phase were absorbed in the collision phase (Fig. 7A). However, the pylon compression amounted to only 14% and 22% of the prosthesis’ (i.e., all components distal to the socket, including the foot) net work in the entire stance and collision phases, respectively. The pylon compression’s negative net work in the entire stance phase (Fig. 7C) corroborated with the pylon compression’s force–displacement profiles (Fig. 7B): both the negative net work and the area under the force–displacement curves showed reducing stiffness led to greater negative net work.

## Discussion

By partitioning the work done by the entire limb, this study assessed the effects of a SAP’s longitudinal stiffness and walking speed on the energetic contributions of the prosthesis and intact joints. We tested the hypotheses that reducing SAP stiffness would: (1) increase the energy stored and returned by the prosthesis, (2) reduce the work demands on the residual knee and hip joints, and (3) that these stiffness effects are more pronounced at faster speeds. We found that the first hypothesis was partially supported in that reducing SAP stiffness increased prosthesis negative work during early stance, but did not affect positive work during late stance. Our last two hypotheses were not supported. Our study adds to the knowledge gained from previous studies using this dataset [23, 32] that with the main effects of reducing stiffness and increasing speed, the prosthesis showed a damping effect (greater energy loss) and led to greater push-off work at the residual hip joint.

Our first hypothesis was partially supported, in that reducing SAP stiffness increased negative work during collision, but did not increase positive work during push-off—implying that the increased energy absorption during collision was not returned. Overall, the prosthesis showed a damping behavior in which the total positive work was less than 13% of the negative work. To further understand the sources of energy within the SAP-integrated prosthesis, we performed additional analyses to isolate the work contribution from the pylon. The pylon compressed mostly during early stance and its reduced stiffness increased the magnitude of negative work at both walking speeds. The pylon returned very little energy later in stance, with positive work less than 0.016 Jkg^−1^ across all conditions, resulting in largely negative net work that resembled the damping effect of the entire prosthesis. However, compared to the net work done by the entire prosthesis, the pylon only contributed ~ 18% of the negative net work done by the prosthesis during stance, suggesting that the compressive pylon does not solely explain the prosthesis’ damping effect. A potential explanation of the prosthesis’ negative net work may be from the components distal to the pylon, specifically, the prosthetic foot, foot shell, and shoes. Although power and work from the prosthetic foot were not directly calculated, we can infer (i.e., prosthesis power minus pylon compression) that the remaining components played a role in the negative work (dissipation) from the prosthesis. In fact, previous bench characterization studies of prosthetic feet and footwear suggest overall damping behavior of these modular components [36, 43].

Our second hypothesis was not supported since there was increased work in the proximal intact joints in certain phases of stance, including greater residual knee negative work during collision and greater residual hip positive work during push-off (despite no change in prosthesis positive work). As the prosthesis produced more negative work during collision with reduced stiffness, the residual knee and hip joints redistributed their work output. Performing an extensor moment during collision, the residual knee joint negative work increased with reduced pylon stiffness, mirroring the greater negative work at the prosthesis albeit at a smaller magnitude work (14–24% of the prosthesis). This finding may indicate that walking with an additional shock-absorbing component from the prosthesis may enhance the natural shock-absorbing function of the residual knee joint. Interestingly, with reduced pylon stiffness, the residual hip performed a flexor moment during collision and positive work increased in a similar magnitude comparable to the knee negative work, essentially opposing the residual knee’s energetics. The functional role of this opposing residual hip-knee work during collision is currently unclear. One possibility is that these results may reflect the actions of multi-articular muscles that span these joints, but identifying specific sources of energy transfer across muscles may need additional computational models [44, 45]. Such musculoskeletal models may reveal insights that are inaccessible with the current study’s analyses, such as how energy is transferred within the lower extremity (including to and from the prosthesis) due to the dynamic coupling of interconnected segments [44, 45]. Altogether, reducing pylon stiffness increased the energy absorption on the total prosthetic limb (summation of prosthesis, knee, and hip) during collision, consistent with a prior study [32].

While reducing pylon stiffness primarily affected the prosthesis work during the collision phase, the residual hip joint work output was altered at various phases of stance, specifically during push-off. Despite no significant changes in prosthesis push-off work (with reduced pylon stiffness), the residual hip joint increased its positive push-off work by 12%. Interestingly, the residual hip joint total positive work did not change across the entire stance phase with varying pylon stiffness, indicating a temporal redistribution of positive work. One potential explanation for this temporal redistribution is that with greater negative work by the prosthesis (and the total limb) during collision, there was a greater demand for positive work later in stance (e.g., push-off) to preserve similar net work (i.e., close to zero) during stance. For example, the prosthesis increased the magnitude of negative work during collision by 0.027 Jkg^−1^ (compared SOFT relative to RIGID stiffness conditions), while the residual hip joint generated 0.012 Jkg^−1^ more positive push-off work to partially make up for the increased energy loss during collision. Currently, it is unclear whether such temporal redistribution of hip work is beneficial to the user, or whether such patterns may affect stability mechanisms, or exaggerate a hip powering strategy during push-off that are common in individuals with lower-limb amputations [46, 47].

While not included in our original hypotheses, we found that reducing pylon stiffness reduced collision work on the sound limb, consistent with a prior study [32]. Individuals with a unilateral amputation typically have greater sound limb collision work compared to healthy control participants [26, 48] and has been implied as a potential risk factor for joint osteoarthritis [49–51]. An association between reduced prosthetic limb push-off work and increased sound limb collision work has been suggested [26], where reduced prosthetic limb push-off would lead to an insufficient redirection of the center-of-mass velocity that leads to greater absorption requirement on the sound limb during the step-to-step transition. Consequently, lower-limb prostheses that contribute to push-off by passively returning elastic energy [9, 49] or through actively generating power [11] have demonstrated reductions in sound limb loading and collision work. We found in our study that the sound limb collision work was reduced by 11–17% with the least stiff conditions, which coincided with greater prosthetic limb collision work and not from increased push-off work. In comparison, a prior study found 22–27% reduction in sound limb collision work when individuals walked (at comparable speeds to our studies) with a powered prosthesis (compared to an unpowered prosthesis) aided by push-off power generation [11]. While the mechanisms of the inverse relationship between prosthetic and sound limb collision work in our study are currently unclear, these results are consistent with a recent study that found prosthetic limb collision work are negatively correlated to sound limb collision [52]. Future studies should examine the role of the prosthetic limb’s negative work on reducing sound limb collision, which may lead to encouraging clinical implications such as preserved health and integrity of intact limb’s joint tissue [50].

Based on the results of this study and previous studies [19, 22, 23, 32], future research may need to consider the potential trade-offs in the benefits and adaptive compensations to support the use and prescription of SAPs. The user may benefit from walking with SAPs as they absorb energy [32] to reduce impact peak forces during early stance [22] with less collision work on the sound limb [32]. In other words, SAPs could assist in weight acceptance on the prosthetic limb, as well as the sound limb. However, walking with SAPs could increase the push-off work demands at the residual hip joint, potentially to balance out the greater negative work done on the prosthesis earlier in stance (collision), which may theoretically increase the metabolic cost [26]. Interestingly, a prior study found that SAPs can reduce metabolic energy cost of walking [53], even though our study found that SAPs absorb more energy. Thus, it is possible that increased prosthetic energy absorption may even reduce metabolic cost, perhaps through decreased collision on the sound limb—but this is merely speculation and further research is needed to unravel the effects of SAPs on the mechanics and metabolic demands of walking. Future studies should also consider the potential benefit of SAPs during tasks that require greater energy dissipation (e.g., downhill walking or stepping down curbs) [19] or in other tasks that require greater attenuation of impact forces (e.g., walking with a weighted backpack) [54–56]. A comprehensive investigation analyzing the effect of SAPs in a wide variety of walking tasks is necessary to support the use and clinical prescription of SAPs.

Potential limitations are specified in this study. The first limitation is the energy lost at the residuum-socket interface cannot be quantified due to our methodology of calculating power of all components below a reference segment (i.e., prosthetic socket) [38]. The second limitation of this study is the power and work profiles of the prosthetic foot and shoes cannot be isolated using the current protocol. However, with our additional prosthesis analysis, we deduced the amount of work produced by the prosthetic foot and shoe combination. Another limitation includes having a short familiarization period (at least 5 min) to properly adjust to each prosthetic condition. Future studies may involve more detailed computational models of the prosthetic components integrated with a muscle-driven model of the intact joints, which could further reveal the energy transfer within and across the prosthesis and the intact muscles.

## Conclusion

In our study, faster walking speeds significantly produced larger magnitudes of mechanical work. Generally, the least stiff SAP (compared to rigid) increased the magnitude of prosthesis negative work during collision by 0.027 Jkg^−1^ (rigid versus least stiff SAP), without affecting the positive work (energy return) later in stance, which implies a damping effect. A portion of this energy loss during collision was partially offset by an increase in the residual hip positive work (0.012 Jkg^−1^ increase in the least stiff versus rigid SAP) later in stance during push-off. We also observed 11–17% reduction in sound limb collision work with the least stiff prosthesis, although the mechanism driving this reduction is currently unclear. While this reduction in sound limb work may imply favorable clinical implications, it is unclear whether the use of SAPs is entirely beneficial during walking. Future studies are warranted to investigate whether SAPs could provide additional user benefits in tasks that require greater energy dissipation (e.g., downhill walking, stepping down curbs) or tasks that require greater attenuation of impact forces (e.g., load carriage, walking on uneven terrain).

## Supplementary Information

**Additional file 1.** Sound ankle–foot power and work. Average power by the sound ankle–foot (n = 12) (A) and mechanical work were computed in the entire stance (B) and during each of the four sub-phases: collision (C), rebound (D), preload (E), and push-off (F). Faster walking speed produced greater magnitudes of work in the entire stance phase (B) and collision (C). Entire stance (B), rebound (D), preload (E), and push-off(F) showed interaction effects. Overall, the sound ankle–foot produced net negative work in the entire stance phase (B). Significant stiffness, speed, and interaction effects are denoted by ‘*’, ‘ + ’, and ‘x’ symbols, respectively.

**Additional file 2.** Sound knee power and work. Average power by the sound knee (n = 12) (A) and mechanical work were computed in the entire stance (B) and during each of the four sub-phases: collision (C), rebound (D), preload (E), and push-off (F). Faster walking speed produced greater magnitudes of work across the entire stance phase (B) and sub-phases (C-F). Preload (E) showed an interaction effect. Overall, the sound limb’s knee produced net positive work across the entire stance phase (B) in all stiffness and speed conditions. Significant stiffness, speed, and interaction effects are denoted by ‘*’, ‘ + ’, and ‘x’ symbols, respectively.

**Additional file 3.** Sound hip power and work. Average power by the sound hip (n = 12) (A) and mechanical work were computed in the entire stance (B) and during each of the four sub-phases: collision (C), rebound (D), preload (E), and push-off (F). Collision (C) showed a stiffness effect. Faster walking speed produced greater work in the entire stance phase (B) and most sub-phases (C-D, F). Overall, the sound limb’s knee produced net positive work in the entire stance phase (B). Significant stiffness, speed, and interaction effects are denoted by ‘*’, ‘ + ’, and ‘x’ symbols, respectively.

**Additional file 4.** Results from sound limb ankle–foot, knee, and hip.

## Data Availability

The data generated or analyzed during this study are available from the corresponding author on reasonable request.

## Abbreviation

SAP: Shock-absorbing pylon
