# Supplementary figures and images for: Reducing stiffness of shock-absorbing pylon amplifies prosthesis energy loss and redistributes joint mechanical work during walking
Source: J Neuroeng Rehabil. 2021 Sep 21;18:143. doi: 10.1186/s12984-021-00939-8 (PMC8456590; doi:10.1186/s12984-021-00939-8)

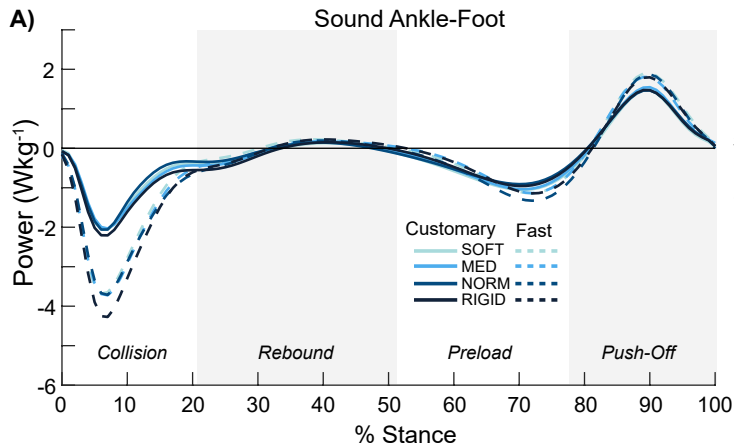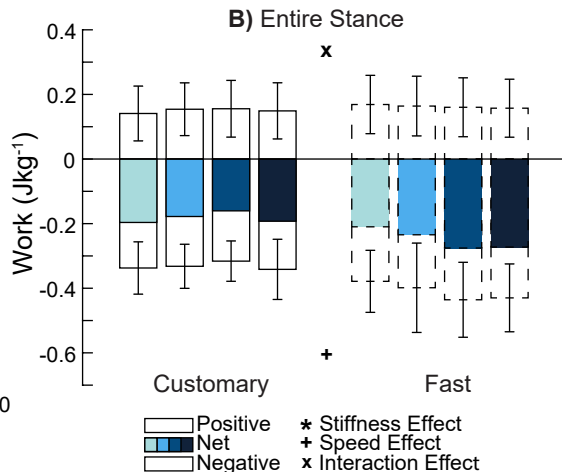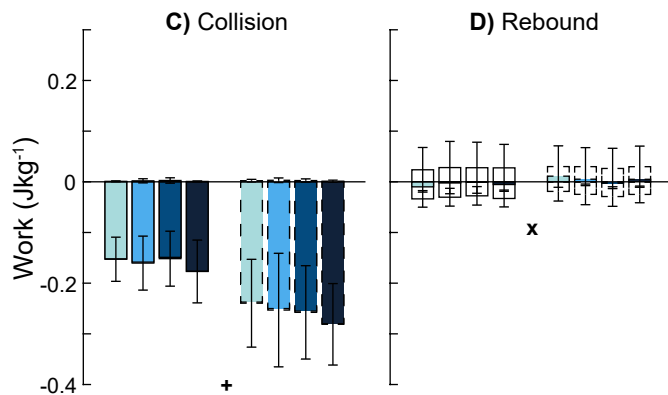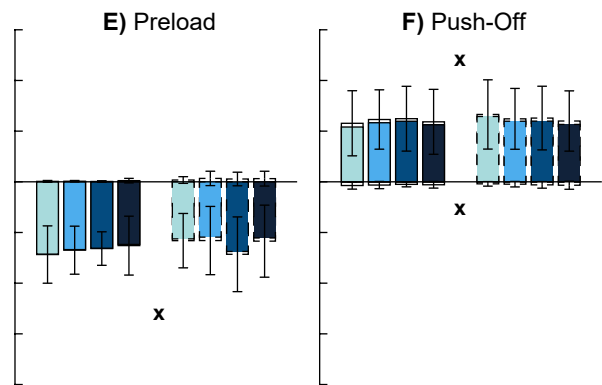

Supplement: Supplementary file 1 — Additional file 1. Sound ankle–foot power and work. Average power by the sound ankle–foot (n = 12) (A) and mechanical work were computed in the entire stance (B) and during each of the four sub-phases: collision (C), rebound (D), preload (E), and push-off (F). Faster walking speed produced greater magnitudes of work in the entire stance phase (B) and collision (C). Entire stance (B), rebound (D), preload (E), and push-off(F) showed interaction effects. Overall, the sound ankle–foot produced net negative work in the entire stance phase (B). Significant stiffness, speed, and interaction effects are denoted by ‘*’, ‘ + ’, and ‘x’ symbols, respectively. [file 12984_2021_939_MOESM1_ESM.pdf]

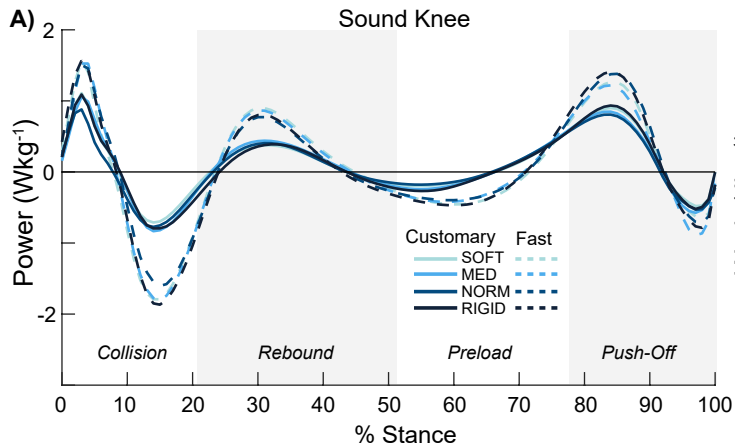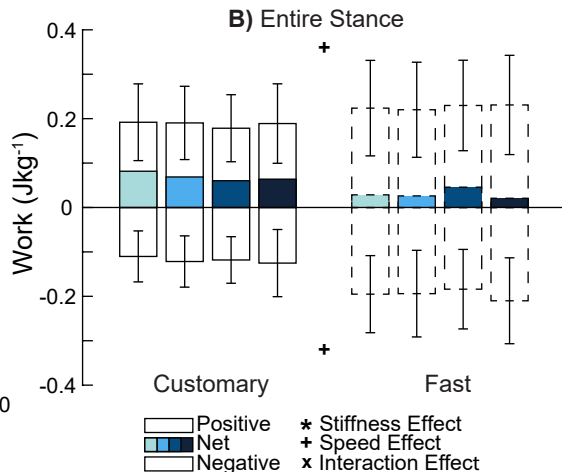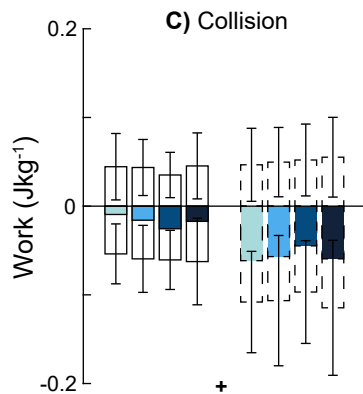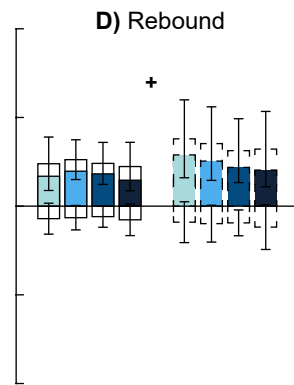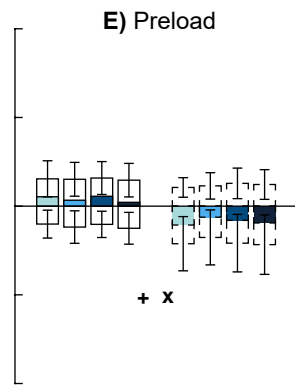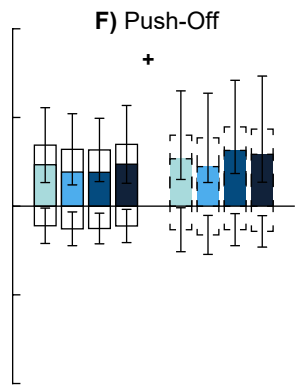

Supplement: Supplementary file 2 — Additional file 2. Sound knee power and work. Average power by the sound knee (n = 12) (A) and mechanical work were computed in the entire stance (B) and during each of the four sub-phases: collision (C), rebound (D), preload (E), and push-off (F). Faster walking speed produced greater magnitudes of work across the entire stance phase (B) and sub-phases (C-F). Preload (E) showed an interaction effect. Overall, the sound limb’s knee produced net positive work across the entire stance phase (B) in all stiffness and speed conditions. Significant stiffness, speed, and interaction effects are denoted by ‘*’, ‘ + ’, and ‘x’ symbols, respectively. [file 12984_2021_939_MOESM2_ESM.pdf]

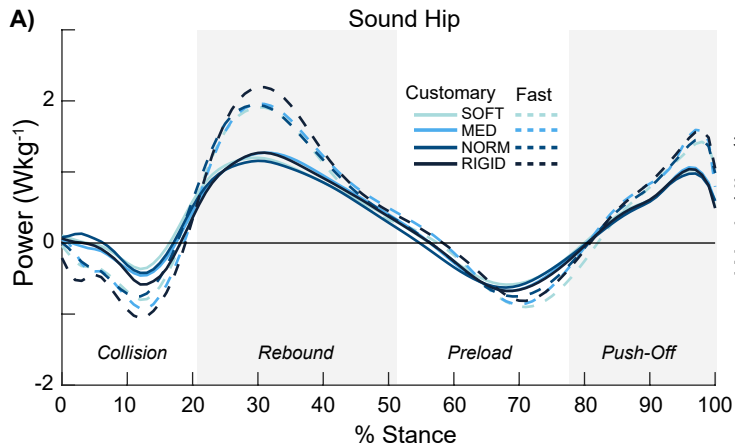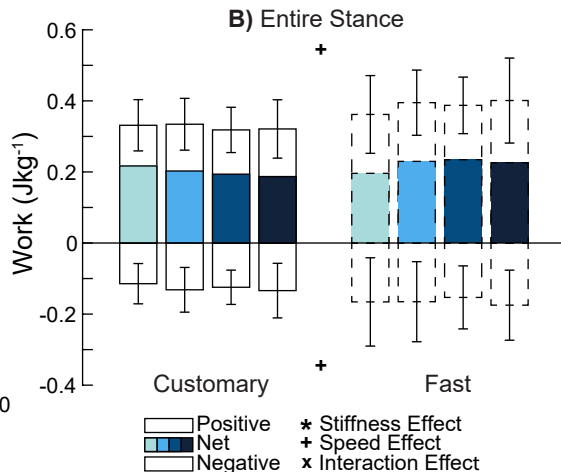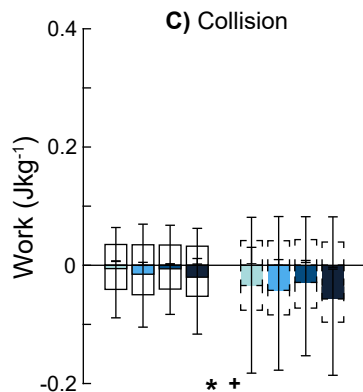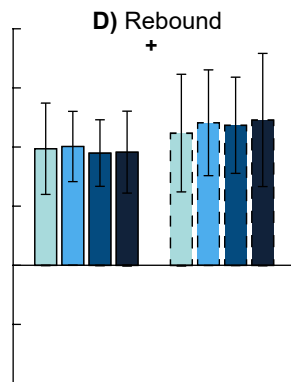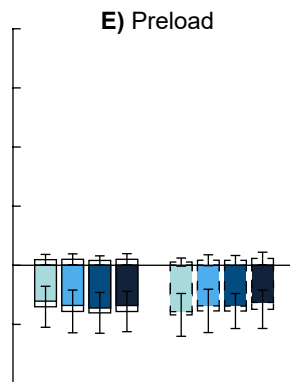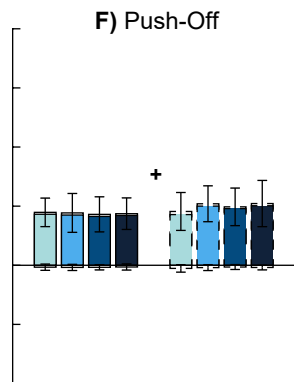

Supplement: Supplementary file 3 — Additional file 3. Sound hip power and work. Average power by the sound hip (n = 12) (A) and mechanical work were computed in the entire stance (B) and during each of the four sub-phases: collision (C), rebound (D), preload (E), and push-off (F). Collision (C) showed a stiffness effect. Faster walking speed produced greater work in the entire stance phase (B) and most sub-phases (C-D, F). Overall, the sound limb’s knee produced net positive work in the entire stance phase (B). Significant stiffness, speed, and interaction effects are denoted by ‘*’, ‘ + ’, and ‘x’ symbols, respectively. [file 12984_2021_939_MOESM3_ESM.pdf]
