# Supplementary material for: Reducing stiffness of shock-absorbing pylon amplifies prosthesis energy loss and redistributes joint mechanical work during walking
Source: J Neuroeng Rehabil. 2021 Sep 21;18:143. doi: 10.1186/s12984-021-00939-8 (PMC8456590; doi:10.1186/s12984-021-00939-8)
Supplement: Supplementary file 4 — Additional file 4. Results from sound limb ankle–foot, knee, and hip. [file 12984_2021_939_MOESM4_ESM.docx]

**Additional File 4**: Results from sound limb ankle-foot, knee, and hip.

**Sound Ankle-Foot:** There were no stiffness effects (p ≥ 0.473, *η*^2^*_p_* ≤ 0.072) in the entire stance phase (Figure S1A-B) and sub-phases (p ≥ 0.057, *η*^2^*_p_* ≤ 0.201, Figure S1C-F).

There was a speed effect for only negative work in the entire stance phase (p = 0.002, *η*^2^*_p_* = 0.595). Fast walking was associated with 24% greater negative stance work compared to customary walking. Apart from the rebound (p ≥ 0.131, *η*^2^*_p_* ≤ 0.195), preload (p ≥ 0.19, *η*^2^*_p_* ≤ 0.151), and push-off (p ≥ 0.343, *η*^2^*_p_* ≤ 0.082) sub-phases, negative collision work showed a speed effect (p < 0.001, *η*^2^*_p_* = 0.778). Fast walking was also associated with 61% greater negative collision work compared to customary walking.

There was an interaction effect (p = 0.046, *η*^2^*_p_* = 0.213) for only positive work in the entire stance phase. Simple main effects analysis revealed no stiffness (p ≥ 0.977, *η*^2^*_p_* ≤ 0.002) and speed (p ≥ 0.443, *η*^2^*_p_* ≤ 0.007) effects. Apart from the collision sub-phase (p ≥ 0.330, *η*^2^*_p_* ≤ 0.094), negative rebound work (p = 0.033, *η*^2^*_p_* = 0.230), negative preload work (p = 0.042, *η*^2^*_p_* = 0.217), positive push-off work (p = 0.017, *η*^2^*_p_* = 0.262), and negative push-off work (p = 0.029, *η*^2^*_p_* = 0.289) showed interaction effects. Simple main effects analysis revealed no stiffness effects (p ≥ 0.591, *η*^2^*_p_* ≤ 0.021) in all the sub-phases and only the negative rebound work showed a speed effect in SOFT stiffness (p = 0 048, *η*^2^*_p_* = 0.044). Fast walking led to 44% less negative rebound work compared to customary walking.

**Sound Knee:** There was no stiffness effect in the entire stance phase (p ≥ 0.074, *η*^2^*_p_* ≤ 0.187, Figure S2A-B) and sub-phases (p ≥ 0.052, *η*^2^*_p_* ≤ 0.206, Figure S2C-F).

There were speed effects for both positive and negative work in the entire stance phase (p ≤ 0.003, *η*^2^*_p_* ≤ 0.789, Figure S2B). Fast walking was associated with 21% greater positive and 65% greater negative stance work compared to customary walking. All the sub-phases showed speed effects (p ≤ 0.009). Fast walking was also associated with increased work: 81% greater negative collision work (p = 0.001, *η*^2^*_p_* = 0.664), 42% greater positive rebound work (p = 0.009, *η*^2^*_p_* = 0.479), 86% greater negative preload work (p = 0.006, *η*^2^*_p_* = 0.517), and 26% greater positive push-off work (p = 0.007, *η*^2^*_p_* = 0.505) compared to customary walking.

There was no interaction effect in the entire stance phase (p ≥ 0.100, *η*^2^*_p_* ≤ 0.074). Apart from the collision (p ≥ 0.053, *η*^2^*_p_* ≤ 0.205), rebound (p ≥ 0.159, *η*^2^*_p_* ≤ 0.143), and push-off (p ≥ 0.115, *η*^2^*_p_* ≤ 0.162) sub-phases, negative preload work showed an interaction effect (p = 0.015, *η*^2^*_p_* = 0.276). Simple main effects analysis revealed no stiffness effect (p ≥ 0.863, *η*^2^*_p_* ≤ 0.008); however, speed effects were observed in SOFT (p = 0.036, *η*^2^*_p_* = 0.049) and NORM (p = 0.045, *η*^2^*_p_* = 0.045) stiffness conditions, where fast walking led to 100-110% greater negative preload work.

**Sound Hip:** There were no stiffness effects in the entire stance phase (p ≥ 0.231, *η*^2^*_p_* ≤ 0.121, Figure S3A-B). Apart from the rebound (p ≥ 0.296, *η*^2^*_p_* ≤ 0.104), preload (p ≥ 0.130, *η*^2^*_p_* ≤ 0.179), and push-off (p ≥ 0.283, *η*^2^*_p_* ≤ 0.151) sub-phases, negative collision work showed a stiffness effect (p = 0.047, *η*^2^*_p_* = 0.121, Figure S1C). One post-hoc pairwise comparison was significant (SOFT-RIGID p = 0.04) where reduced stiffness produced 22% less negative collision work.

There were speed effects for both positive and negative work in the entire stance phase (p ≤ 0.036, *η*^2^*_p_* ≤ 0.722). Fast walking was associated with 18% greater positive stance work and 31% greater negative stance work compared to customary walking. Apart from the preload sub-phase (p ≥ 0.251, *η*^2^*_p_* ≤ 0.118), the rest of the sub-phases showed speed effects (p ≤ 0.025). Fast walking was also associated with increased work: 78% greater negative collision work (p = 0.019, *η*^2^*_p_* = 0.409), 22% greater positive rebound work (p = 0.002, *η*^2^*_p_* = 0.615), and 14% greater positive push-off work (p = 0.025, *η*^2^*_p_* = 0.379) compared to customary walking.

There were no interaction effects in the entire stance phase (p ≥ 0.077, *η*^2^*_p_* ≤ 0.185) and sub-phases (p ≥ 0.053, *η*^2^*_p_* ≤ 0.205).
